# How Established Organizations Combine Logics to Reconfigure Resources and Adapt to Marketization: A Case Study of Brazilian Religious Schools

**DOI:** 10.1177/0022243721999042

**Published:** 2021-07-13

**Authors:** Pierre-Yann Dolbec, Rodrigo B. Castilhos, Marcelo J. Fonseca, Guilherme Trez

**Keywords:** conformity, differentiation, education, institutional logics, marketization, resource-based theory

## Abstract

Marketization—the entry of the market logic into a field originally insulated from it—is a transformative force that has reshaped many fields, including education, health care, the arts, and religion. Marketization brings a unique set of challenges for established organizations: it opens a field to market-style mechanisms of consumer choice and competition, which undermines the legitimacy of established organizations, and it creates contradictory demands for organizational actions. How can established organizations adapt to marketization? To answer this question, the authors study the adaptation of five established religious schools to the marketization of education in Brazil. They develop the novel hybridization strategy of nested coupling and explain that established organizations respond to marketization by balancing competing demands for differentiation and conformity. The authors show how religious schools nest the market logic within the religious logic by reconfiguring their resources to conform to market demands while differentiating themselves through their religious orientation. Nested coupling provides a novel strategic approach for established organizations in marketized or marketizing fields, such as hospitals, museums, and schools, to capitalize on a logic that preexists marketization and to create a unique competitive positioning in the market.

Marketization is a transformative force that has reshaped many fields, including the arts, education, health care, and religion. During marketization, the dominant logic of a field—“the socially constructed, historical pattern of material practices, assumptions, values, beliefs, and rules” (Thornton and Ocasio 1999, p. 804)—shifts to the market logic. As a result, organizations that preexisted marketization and were initially insulated from market forces, which we refer to as “established organizations,” must adapt to “the ideology, goals, and principles of the modern market” (Fırat 2020, p. 21).

Established organizations in a marketizing field face a stark shift. In nonmarketized fields, the dominant logic, supported by government mandates, laws, and widely diffused norms, confers authority to professionals and their organizations. They determine what products and services are perceived as proper, desirable, or appropriate by stakeholders—that is, what is legitimate (Suchman 1995). There is thus little need to be market oriented. For example, prior to marketization, museums and hospitals reflected art and medical professionalism logics, which situated the main source of legitimacy with museum curators and physicians (Battilana, Leca, and Boxenbaum 2009; Eräranta et al. 2020). Consequently, museum curators decided on exhibitions, and physicians decided on patient treatments.

Marketization creates a unique set of challenges for established organizations. First, it opens a field to “market-style mechanisms of consumer choice and competition among providers” (Lubienski 2005, p. 464), which undermines the legitimacy of established organizations and threatens their long-term survival (Handelman and Arnold 1999). In a marketizing field, consumers increasingly drive what products and services are perceived as proper, desirable, or appropriate. They expect their demands to be satisfied (Johnson, Anderson, and Fornell 1995). Satisfying customer demands thus becomes an important source of legitimacy (e.g., Kates 2004), and established organizations must address consumer needs and pressures that arise from competitors striving to do the same, or otherwise face a loss in legitimacy. Second, the market logic is typically in conflict with the logic(s) that preexisted marketization, creating contradictory demands for organizational actions (Greenwood et al. 2011). As a result, established organizations that embrace the market logic to better address customer needs and competitive pressures face internal conflict, which they must address (Besharov and Smith 2014). This situation is unique to established organizations, as new contenders are unconstrained by a prior logic and can fully embrace the market logic (e.g., for-profit schools in education).

How can established organizations successfully adapt to marketization? A likely answer from marketing would be to adopt a market orientation. Market orientation—the defining strategic orientation in marketing—focuses on addressing customer demands and responding to competitive pressures. It is a strategic orientation aligned with the market logic (Faria 2015). Alternative strategic orientations from the marketing literature also aim at making a firm more competitive by abiding by the market logic by focusing on production, sales (e.g., Noble, Sinha, and Kumar 2002), entrepreneurship, or technology (e.g., Zhou, Kin, and Tse 2005).

In fact, many established organizations that face marketization do become at least partly market oriented. For example, following marketization, museums might stage popular and entertaining exhibitions to attract visitors (Eräranta, Moisander, and Penttilä 2020), and hospitals might treat patients as customers who are provided treatment choices (Tritter 2009). However, increasingly catering to the market is at odds with the logic(s) that drive established organizations. The incompatibility between the market logic and the logics that preexist marketization makes adopting such strategic orientations problematic and can impede organizational adaptation (e.g., Press et al. 2014).

Work in marketing does not consider how firms can manage incompatible institutional logics or offer an intermediate solution for strategic adaptation: firms either embrace a new strategic orientation aligned with the market logic (Gebhardt, Carpenter, and Sherry 2006) or refuse to do so (Press et al. 2014). Thus, it is unclear how established organizations that risk internal conflict if they embrace the market logic—and yet need to respond to the market to ensure their survival—can adapt to marketization.

To examine how established organizations can respond to both the market logic and a preexisting logic while minimizing internal conflict, we adopt an institutional logics perspective. From this lens, firms address competing institutional logics through hybridization—that is, incorporating elements from contradictory logics within an organization (Battilana and Dorado 2010).

Two assumptions dominate existing work on hybridization. First, firms hybridize to conform to conflicting institutional logics, such as microfinancing firms that help the poor (i.e., conforming to a “development logic”) but do so profitably (i.e., conforming to a “banking logic”) (Battilana and Dorado 2010, p. 1419). Second, combining conflicting logics creates internal tensions. As a result, existing work has concentrated on how firms manage tensions to conform to conflicting logics (for reviews, see Battilana, Besharov, and Mitzinneck [2017] and Greenwood et al. [2011]). Hybrids do so in two distinct ways: they either separate logics by, for example, compartmentalizing strategic responses into different functional units (e.g., athletic departments at universities in Kraatz and Block [2008]) or integrate logics by creating new organizational forms (e.g., microfinancing firms in Battilana and Dorado [2010]).

Although hybridization helps explain how established organizations can manage internal tensions to conform to competing institutional logics when adapting to marketization, it does not solve how such organizations can differentiate themselves from competitors and create superior value for consumers.

In summary, established organizations facing marketization need to respond to a unique set of challenges, but prior work does not provide guidance on how they can do so. To resolve this gap and answer our research question, we study the adaptation of five established religious schools to the marketization of education in Brazil. We combine institutional logics with resource-based theory, which is ideal for explaining how organizations balance demands to conform to competing logics while differentiating themselves from competitors and creating superior value for consumers: the first caters to conformity, while the latter informs differentiation and value creation. We develop a novel hybridization strategy—nested coupling—that explains how established organizations respond to the market logic by transforming their resources to create value for customers and differentiate themselves while minimizing internal conflicts.

Our contributions are as follows. First, we introduce nested coupling as a novel hybridization strategy and show how it leads established organizations to respond to marketization. In contrast to prior work that mostly concentrates on conformity, we demonstrate how nested coupling leads organizations to balance demands for conformity and differentiation. To do so, we combine resource-based theory with institutional logics, which offers a new theoretical approach to study organizations and marketing strategy. Second, we expand on nested coupling to provide theoretical insights into strategic orientation. We propose that organizations should implement strategic orientations differently depending on the dominant logic of their field. We then discuss implications for hybrid strategic orientations. We exemplify our ideas using market-driving and market-driven orientations and show how nested coupling informs a hybrid driven–driving orientation. We now turn our attention to our theoretical framework.

## Combining Institutional Logics and Resource-Based Theory

Firms differentiate themselves and achieve conformity by developing and exploiting resources—the “assets, capabilities, organizational processes, firm attributes, information, knowledge, etc.” controlled by a firm that enable value-creating strategies (Barney 1991, p. 101).

The value of resources depends on their fit with the institutional environment (Oliver 1997), which we operationalize as an alignment between resources and the prescriptions of the institutional logic(s) governing a field (e.g., Ertimur and Coskuner-Balli 2015; see also Zhao et al. 2017). By logic prescriptions, we refer to the assumptions, values, and beliefs of an institutional logic, which prescribe how organizations should act. Resources can be valuable because they help an organization conform to the prescriptions of a logic, differentiate itself from competitors, or both (see Zhao et al. 2017). Conforming to logics provides legitimacy, while differentiating from competitors addresses competitive pressures. Firms need to balance these demands for conformity and differentiation and strive to be “as different as legitimately possible” (Deephouse 1999, p. 147).

To conform to institutional logics and differentiate themselves from competitors, firms develop, bundle, and exploit resources. A single resource rarely allows a firm to do so, and firms usually create value by bundling resources in unique ways (Sirmon et al. 2007). Whether a resource can be used to achieve conformity or differentiation depends on *what* the resource is and *how* it is put to use to address prescriptions (see Grant 1991; Oliver 1997).

During marketization, the dominant logic of a field shifts to the market logic. This shift changes the prescriptions that structure organizational action (e.g., Eräranta, Moisander, and Penttilä 2020; Molnar 1996) and what is perceived as valuable (Ertimur and Coskuner-Balli 2015; Zhao et al. 2017) and creates new market-based competitive pressures (Lubienski 2005). Shifts in logics such as marketization can be difficult for established organizations because they have developed resources that reflect the prescriptions of the logic that governed their field prior to the entry of a new logic. For example, the organizations we study developed valuable resources for the better part of the last hundred years by responding to religious prescriptions. A shift to the market logic transformed the field’s prescriptions and negatively affected the value of the organizations’ resources because they could not conform to market prescriptions, which undermined their legitimacy; however, the organizations also possessed resources different from those of a new crop of market-oriented competitors, which presented opportunities for differentiation. We explain these core ideas of our theoretical framework through the following example from the marketizing field of education.

A religious school owns a chapel on its school grounds. The chapel (i.e., *what* the resource is) can inherently be a source of conformity or differentiation depending on whether it aligns with the institutional logic that governs the field. Under the religious logic, the chapel allows the school to conform to prescriptions of spirituality and religious practice. However, in a marketized educational context where the market logic dominates, such a religious asset does not contribute to conformity. It might, though, present an opportunity for differentiation, because market-oriented competitors are unlikely to possess such a resource.

The role of the chapel in supporting conformity or differentiation can be transformed depending on how it is used. This is particularly important for established organizations in a transforming field such as in our context, as resources that do not readily meet the expectations of a new dominant logic can be transformed or bundled with other resources to do so (i.e., *how* they are used). For example, in the marketized field of education, the prescriptions of the religious and market logics are in contradiction. The chapel does not conform to market expectations, but it presents an opportunity for differentiation. The school could take either of the following avenues. It could use the chapel to strengthen its differentiation from market-oriented schools. For example, it could bundle the chapel with other religious resources, such as teachers who are also priests or capabilities associated with celebrating Mass. Alternatively, it could transform the use of the chapel to conform to market prescriptions. For example, under the market logic, it is valuable for schools to develop students’ emotional intelligence (Davies and Bansel 2007). Instead of holding Mass, the school could hold nondenominational spiritual celebrations that enable students to connect with themselves and others, contributing to the development of their emotional intelligence and transforming the chapel into a source of differentiation that also conforms to expectations of the market logic. Should the religious school reinforce its religious orientation or adapt to the market logic? Nested coupling informs how established organizations facing marketization can take such strategic decisions.

How firms manage the integration of competing prescriptions depends on the compatibility of prescriptions and whether their integration is managed within the core or at the periphery of the organization. We define compatibility as the coherence between prescriptions of competing institutional logics (see Besharov and Smith 2014). For example, the prescription of religious communion from the religious logic might be compatible with that of emotional development from the market logic, which would facilitate their integration. We draw from Hannan and Freeman (1984) to differentiate between the organizational core and periphery. The organizational core is composed of the following hierarchically organized elements: organizational goals, forms of authority, core technologies (broadly defined as infrastructure, techniques, methods, and processes used to create value), and marketing strategy (see Hannan and Freeman 1984, p. 156). Compatibility and core versus periphery are important because they influence the likelihood of internal tensions when managing competing logics (Besharov and Smith 2014; Gümüsay, Smets, and Morris 2020). We expand on how this occurs in our findings.

In summary, we combine institutional logics with resource-based theory to propose that established organizations develop resources in alignment with the logic that governed their field at a certain point in time. Marketization shifts the dominant logic of a field to the market logic, which brings new market-style mechanisms of consumer choice and competition and negatively affects established organizations by undermining their legitimacy and introducing competitive pressures. Organizations must thus address their lack of conformity to market logic while differentiating themselves from competitors. Integrating the market logic is problematic, though, because its prescriptions contradict those of the preexisting logic. These contradictions can elicit internal tensions, which vary depending on the compatibility of prescriptions and whether they are managed in the organizational core or periphery. Next, we explain our context and expand on our methodology.

## Context and Methodology

To contextualize our research, we first identify the institutional logics that orchestrate the Brazilian education field: a public, religious, and market logic. We follow a “pattern matching” approach, a well-acknowledged method in which researchers create ideotypical logics from extant literature (Reay and Jones 2016) and then match data to each ideal type. References for the scholarly research we analyzed and cite to create the three ideotypical logics and support our findings are provided in Web Appendix A. These logics are interinstitutional (i.e., they structure society as a whole; see Thornton, Ocasio, and Lounsbury 2012).

### Institutional Logics in Education

Christianity is the historical starting point of education in the Western world. Education orchestrated by the religious logic serves the Church. Its mission is to form faithful and fraternal people to properly participate in a religiously structured society (Grace and O’Keefe 2007). Religious education is based on the principles of a person’s innate goodness, sacramentality, community, and spiritual experience (Groome 2005).

Public education emerged with the industrial revolution (Katz 1976). Under the public logic, education serves a nation. It is positioned as a universal right based on principles of equality of opportunity and accessibility to all (Nasaw 1979). Its goal is to train workers and politically compliant citizens to support the growth of a nation (Katz 1976). Education is centered on the development of the student as a productive member of society, which commonly translates into standardized training to teach skills and transmit moral values (Nasaw 1979).

The marketization of education refers to the spread of market forces in education, which happened with neoliberalism’s rise to dominance in the 1970s (Baltodano 2012; Harvey 2005; Molnar 1996). Under the market logic, education serves the market. Education is centered on the development of students as entrepreneurial actors who learn the skills that can propel their careers. However, education is not equally accessible to all: it is based on competition, meritocracy, economic rationality, and self-responsibility (Baltodano 2012) and can be restricted on the basis of achievements or tuition. Table 1 summarizes the characteristics of each institutional logic.

**Table 1.** Prescriptions of the Ideotypical Logics Governing Education.

**Dimension**	**Religious^a^**	**Public^b^**	**Market^c^**
Role of education	To pursue God’s grace, a mean for evangelization	A universal human right, a means to support the nation	A commodity, a means to sustain the market economy
Foundational principles	Human goodness, sacramentality, community, spiritual autonomy	Equality of opportunity, universality, and public obedience	Competition, merit, economic rationality, and self-responsibility
Organizational mission	Forming faithful and fraternal people to participate in a religious society	Forming politically compliant citizens to participate in a democratic society productively	Forming self-responsible and entrepreneurial students to participate in the job market
Educational praxis	Develops spiritual, intellectual, and emotional dimensions	Develops cognitive skills and moral character	Develops competitive drive, hard and soft skills
Sources of authority	Religious affiliation and recognition by a religious authority	State recognition, educational experts and pedagogical consultants, policy makers	School rankings, cost competition, differentiation, customer needs
Primary actors	Clergy members, ordained ministers, and religious educators	Teachers, principals, managers, school boards, policy makers	Consultancy firms, private enterprise managers as school managers
Source of financing	Religious organization, philanthropy, public subsidies, tuition	Philanthropy, public funding	Philanthropy, public subsidies, tuition
Accessibility	Inclusive within religious-based boundaries	Inclusive, universal	Exclusive; tuition- and performance-based

^a^ Based on work on religious education (e.g., Fuchs 2017; Grace and O’Keefe 2007).

^b^ Based on work on public education (e.g., Katz 1976; Nasaw 1979).

^c^ Based on work on marketized education (e.g., Baltodano 2012; Molnar 1996).

*Notes:* The full list of works used to create these ideal types is referenced in Web Appendix A.

Each institutional logic brings competing assumptions, values, and beliefs, which prescribe how organizations should act. A shift in logics changes prescriptions. For example, in education, the market logic challenged the public logic. Rather than being conceptualized as a common good, education became a source of competitive advantage for students. Education was privatized, and the profit-seeking drive of private organizations enhanced competitive dynamics to bolster student enrollment—now viewed as a source of income. This led to diminishing investments in the humanities and arts, disciplines for which there is less occupational opportunity (and thus less enrollment), in favor of profit centers such as business and engineering schools, which grant marketable degrees. Curricula were standardized to manage costs. Organizations started to invest heavily in advertising to attract students. To improve organizational performance, they borrowed from business practices, methodologies, and approaches and concentrated on certifications and accreditations and national and international rankings (see Baltodano 2012; Harvey 2005; Molnar 1996). Next, we explain how these three logics interacted during the evolution of the Brazilian education market.

### The Evolution of the Brazilian Educational Market

Education in Brazil has its roots in the country’s colonial period. Since then, the market has gone through three logic-based stages. From the late 1500s to the early 1900s, education was mostly orchestrated by the religious logic. The Jesuits arrived in the country with Portuguese colonizers and founded the first schools. In this early period, the main goals of schools were to evangelize the Indigenous population and to educate the children of the first colonizers under Catholicism (Klein 2016).

In 1759, a reform reinforced the power of the crown and the nascent state. This marks the emergence of public logic. As part of the reform, the Jesuits were expelled from the country, and their schools were transferred to the state. Pedagogical thinking became inspired by Enlightenment ideals “at the service of the political interests of the State” (Maciel and Neto 2006, p. 470). In 1824, Brazil’s new constitution guaranteed free elementary education to all citizens, and education’s main goal became the formation of citizens. This first attempt at nationalizing education failed, and the Jesuits returned in 1841 (Maciel and Neto 2006).

Around the same time, the arrival of new religious groups expanded religious-based education, further differentiating free public schools governed by the state from private (but not-for-profit) religious schools. This divided the field into two segments: public and private. Most of Brazil’s population was educated through the public system, whereas religious private schools catered to Brazil’s elite (Akkari 2013). We concentrate on the latter market segment.

In the late 1980s and early 1990s, the country underwent neoliberal reforms, which transformed the private education market. Regulative changes opened the door to for-profit schools (Brazil Casa Civil 1988) and established common grounds for education nationwide (Brazil Casa Civil 1996). Private schools continued to cater to the elites. In 2006, 80% of students belonged to the highest income bracket and 5% belonged to the lowest (Curi and Menezes-Filho 2009). New market-oriented competitors positioned themselves around high performance on national ranking tests and future employability.

Since then, the private education market has further segmented into three market categories: a first category composed of religious organizations; a second category composed of large educational groups that offer a low-cost, standardized education (Carbonari 2015); and a third category composed of elite, market-oriented schools that deliver a high-quality but expensive educational experience. This last type is characterized by innovative learning experiences and an international, language-oriented curriculum (Rasmussen 2019). These latter schools compete directly against the organizations we examine.

Religious schools began to adapt in the mid-2000s. Some took the route of cost reduction and standardization to compete in the low-cost market segment. Others, such as those we study, adapted to compete against elite, market-oriented schools.

### Data Collection and Analysis

Given our theory-building perspective, we chose a case study design and concentrated on a set of organizations, consistent with existing research (e.g., Dalpiaz, Rindova, and Ravasi 2016). We answer our research question through an inductive analysis of five educational organizations of the Brazilian Jesuit Educational Network (BJEN). The BJEN is a network of 17 schools located across Brazil. The Brazilian Jesuit Order created the BJEN in 2014 to respond to changes in the educational market. BJEN schools have between 60 and 150 years of history. Until recently, they were positioned as market leaders in their local contexts, a position that was threatened as the market logic gained dominance.

We collected data from 2017 to 2020. Our data cover changes in organizations from 2014, after the creation of the BJEN. In addition to data on our five organizations, we collected data on BJEN. Our data set is composed of interviews, internal documents, and field notes based on participant observation conducted during meetings that informed the strategic reorientation of the five schools and BJEN. Table 2 summarizes our data and details the organizations. We use pseudonyms in place of the real names of the organizations.

**Table 2.** Profile of Organizations and Data.

**Cases**	**Christ School**	**Mary School**	**Apostle School**	**Jesus School**	**Priest School**
Founding year	1905	1957	1890	1956	1903
Number of students in 2016	2,300	2,765	3,000	2,000	2,800
City population (millions) in 2016	.5	1.7	1.5	.6	6.5
Net student acquisition (%) in 2016	−4%	−22%	−1%	4%	0%
ENEM position in 2015 (Brazil)^a^	822	394	423	233	42
ENEM position in 2015 (City)^a^	7	8	9	3	6
Market share in 2011^b^	9.6%	2.6%	5.0%	6.6%	.9%
Market share in 2019^b^	5.9%	1.8%	4.3%	7.1%	.7%
**Data Collection (Schools)**	**Christ School**	**Mary School**	**Apostle School**	**Jesus School**	**Priest School**
Interviewed managers	8	8	6	5	3
Interviewed teachers and coordinators	7	3	5	3	0
Interviewed experts	4	4	3	4	3
Interviewed parents	15	10	17	14	13
Internal documents (pages)	5 (28)	7 (64)	11 (101)	3 (18)	1 (2)
**Data Collection (Network)**	**BJEN**				
Interviewed head managers	2				
Interviewed board members	4				
Internal documents (pages)	14 (≅470)					

^a^ ENEM (National Exam of High School). National ranking based on 15,000 schools (https://bit.ly/2y76jfN).

^b^ Internal data (based on the Census of National Education).

We entered the field in 2017, when three of the four authors were invited to take part in a multiyear consulting project to generate market knowledge on the positioning of the BJEN schools, which gave us access to organizational members and customers. From the outset, the organizations consented to participate in a scholarly research project. We conducted interviews with a total of 135 experts, managers, teachers, and parents of current and prospective students of each organization, representing about 1,800 single-spaced pages of transcribed material. We obtained participants’ consent and informed them that they might be quoted in an academic article. Interviews greatly varied in length, from 15 to 130 minutes. We first interviewed key personnel for each organization. In subsequent interview rounds, we concentrated on organizational members best positioned to inform our emergent findings. Interviews with experts addressed market transformations and the challenges faced by religious organizations. Interviews with managers covered perceived changes and challenges in the market and the schools’ responses. We interviewed teachers to understand how they implemented changes in practice. Interviews with parents dealt with their expectations regarding education, their choice criteria, and their perceptions of and satisfaction with the schools. Drawing on emergent findings, we conducted additional interviews with managers and teachers of the five organizations as well as with BJEN managers and board members. We also collected different types of strategic documents from the BJEN and the five schools that explained how the organizations functioned and their strategic efforts to adapt. Finally, in the context of our consulting project, we engaged in participant observation during meetings with the management teams of the BJEN and the organizations. Participant observation allowed for a better understanding of how different logic prescriptions informed strategic planning and decision making.

Our data analysis was performed iteratively with our data collection, which is a standard procedure in qualitative research (Belk, Fischer, and Kozinets 2013). The first two authors coded the data set independently. We coded our data using both bottom-up and top-down approaches (i.e., we inductively generated codes based on the data, and we used concepts related to our conceptual framework to inform our coding). We first coded within organizations and then compared across them.

From the start, we saw this context as fruitful to explore how organizations adapt to a shift in institutional logics: we knew that the organizations were transforming their strategies to respond to marketization. To support our analysis, we created ideotypical logics based on a review of the academic literature (Reay and Jones 2016; for an example, see Dalpiaz, Rindova, and Ravasi 2016). At this stage, we also coded our data set from the ground up by first generating emic, first-order codes (e.g., “active learning methodologies”). We then combined first-order codes into second-order themes (e.g., “transformation of learning experience”) and revisited our data set by coding from a theoretical perspective.

We made sense of our second-order themes through iterative cycles between reviewing the literature and refining our analysis, which led us to abstract them into four mechanisms that constitute nested coupling. To better inform emerging themes and respond to reviewers’ recommendations, we conducted additional interviews on how the schools performed hybridization in practice, the rationale behind the different approaches used to integrate institutional logics, and the reasons for the concomitant reinforcing of responses to the previous logic with adaptations to the new one. We concluded our project by obtaining feedback from a top manager of the BJEN, who confirmed our findings and provided additional details.

## How Established Organizations Respond to Marketization

Our findings explain how established organizations respond to marketization. To do so, they reconfigure their resources to manage competing logic prescriptions and balance demands for conformity and differentiation. To position the strategic response of religious schools to marketization, the first section of our findings expands on changes in their competitive environment. In our second section, we summarize key strategic insights associated with nested coupling and present the four mechanisms constituting this novel hybridization strategy.

### Changing Institutional Logics as a Threat to Firms’ Competitive Advantage

Before the emergence of the market logic, the organizations were positioned around tradition, spirituality, and excellence in developing students’ critical thinking and humane values. This positioning resulted from resources developed to respond to religious prescriptions such as a conception of education as holistic (i.e., training students by engaging their minds, body, and spirit). Conforming to the religious logic bestowed legitimacy and authority to the organizations, which were perceived locally as elite schools that had educated several prominent politicians, doctors, and businesspeople. A Priest School manager explains this as follows:

Being here for 115 years is not random. We have a strong tradition and a very strong reputation in the city. Many of our alumni have already made history in the city. You can easily find them…whether at a law firm, a clinic, or an office; you will always find a former student who knows the school, who has family members who studied here. These professionals grant a reputation to the school that is based on educational excellence. (Peter, General Manager)

However, marketization brought forth market-style mechanisms of consumer choice and competition, which unsettled religious prescriptions from which the organizations’ resources derived their value and devalued the schools’ resources. Managers started to notice that “people think that what is traditional is retrograde, outdated” (Danton, Academic Director, Apostle School). Parents and students gained influence to orient choice criteria, and satisfying customer demands became an important source of legitimacy. Families started to “expect a more dynamic, innovative school. They want a different model of teaching and learning, not the traditional one” (Danton). Choice criteria became oriented around heightened employability after graduation, school rankings based on national standardized tests, and internationally oriented curricula (see Table 1). The organizations acknowledged the devaluation of existing resources that conformed to religious prescriptions and the need to address new market prescriptions:

The idea that our humanizing project is enough doesn’t support us anymore. The families, our clients, invest in our humanizing proposal. But, at the end of the day, they really want their children to get accepted into medicine; they want the younger kids to have very good scores on the Brazil Test [elementary school achievement test]. Today, they arrive at the enrollment meeting asking what our ENEM [high school achievement test] ranking is. (Loana, General Coordinator Primary School, Christ School)

Moreover, marketization brought new competitive pressures to the field, which further complicated the schools’ situation. For example, market-oriented schools were advertising heavily, where little advertising existed prior. To better their position on national rankings, market-oriented schools also poached top students from competitors. Peter (Philosophy Teacher, Christ School) comments, “They find out who our best students are and offer them scholarships.”

However, the organizations could not simply adapt to marketization by embracing the market logic. They had already spent decades developing their resources to respond to religious prescriptions. In addition, some market prescriptions were antithetical to religious ones, which is perhaps best exemplified by frequently mentioned disappointments with the marketization of education by the managers we interviewed. For example, Father Santos (Top Manager, BJEN) laments that “ultimately, everything is turning to capitalism…which transformed education into a commodity.” Father Mark (Top Manager, BJEN) adds that a sole focus on “job placement as a vehicle to form the labor force and to feed capitalism forgets about human beings.” This marketized view of education is in tense opposition with the educational philosophy of these schools, which responds to religious prescriptions and is orchestrated around delivering integral formation aimed at holistic development (see Table 1).

Consequently, the organizations faced a conundrum. They needed to conform to both religious and market prescriptions, which were sometimes in opposition, while reinforcing their competitive positioning. The following conversation between two Apostle School managers exemplifies this problem:

Ivonne, Learning Coordinator: One of our strengths is that we are well-recognized as a school that provides a human Jesuit formation, and regardless of everything, what has always remained, what has always been the guiding thread, is respect for the student, his development as a person, and the permanent pursuit for human excellence.

Maia, Marketing Supervisor: But this is a challenge for us. We have a strong image as a Jesuit school, but they [parents and students] want us to be innovative and want us to keep being us.…At the same time, parents are concerned about challenges [of job market competition] and new trends in education but want something that looks like us with some traditional values.

It took time for the schools to adapt to market prescriptions. Managers initially “refused” to respond to market prescriptions because they saw “the market as the voice of capitalism and [economic] liberalism” (Father Santos). By the early 2010s, more than a decade after marketization had started, the schools’ competitive positioning had worsened. Forced to engage with the market, managers began to recognize “that some interesting things were coming from some market[-oriented] schools––things that they were ahead of us in––and that being attentive to the market does not necessarily mean giving up our values, on the contrary” (Father Mark). To support their transformation, the schools formed the BJEN in 2014 to “reinforce our strengths.…So, we accepted the new market-based context of education so we could learn to optimize our costs and to position ourselves better” (Father Mark). Next, we describe how they pursued this transformation to conform to the market logic while preserving their alignment with religious prescriptions and addressing new competitive pressures.

### Nested Coupling

Nested coupling refers to the subordinate integration of a competing set of prescriptions through resource reconfiguration. In our context, this creates a hierarchical blending of the religious and market logics where the religious logic informs the integration of the market logic. This hybridization strategy allows organizations to conform to competing prescriptions while responding to competitive pressures (i.e., to balance demands for conformity and differentiation). Nested coupling is constituted of four mechanisms—core subsumption, core integration, peripheral integration, and peripheral separation—that explain how organizations integrate competing prescriptions by reconfiguring their resources.

Risks of internal tensions inform the management of competing prescriptions. Within the core, incompatible market prescriptions present high risks of internal tension, which leads the organizations to subsume them within religious prescriptions. Compatible market prescriptions present lower risks of tensions, which leads to their integration with religious prescriptions. In both cases, organizations manage prescriptions by reconfiguring their resources to improve their relevance to new market demands while retaining their engagement with the religious logic. This is important because resources that can support differentiation still need coherence with dominant prescriptions (i.e., they need some conformity to be perceived as valuable; Zhao et al. 2017).

At the periphery, tensions are less likely to emerge. The organizations benefit from more strategic latitude, which they use to separate prescriptions and respond to them fully. They also integrate prescriptions by adapting and developing resources and bundling them with those of third parties to strengthen responses to market prescriptions and by capitalizing on existing resources to reinforce responses to religious prescriptions. Peripheral strategic responses address the limitations of responses within the core. Table 3 summarizes our findings.

**Table 3.** Nested Coupling: Summary of the Mechanisms and Evidence.

**Mechanism**	**Prescription**	**Examples of Resources Deployed, Developed, and Reconfigured**	**Outcome**	
*Core subsumption*: Reconfigure resources to absorb incompatible market prescriptions within the core	*Low compatibility*: Holistic education (REL) vs. test-oriented education (MKT)	*Educational philosophy*: Educational goals, ECP (strategic plan), learning maps, curricula, employees’ expertise, teaching activities	*Mostly differentiation*: Reconfigures curricula to reinforce the schools’ holistic educational philosophy while absorbing demands of academic performance	

*Core integration*: Reconfigure resources blend compatible market prescriptions within the core	*High compatibility*: Jesuit autonomy (REL) vs. self-responsibility (MKT)	*Teaching methodology*: Educational goals, ECP, learning maps, schools’ curricula, teaching methodologies, employees’ expertise	*Differentiated conformity*: Reconfigures teaching methodologies to integrate active learning and form self-responsible students with religious ideals	

*Peripheral integration*: Reconfigure resources to blend compatible market prescriptions at the periphery	*High compatibility*: Religious charity (REL) vs. forming employable students (MKT)	*Volunteering extracurriculars*: Educational philosophy, educational goals, schools’ history, schools’ network; employees’ expertise, student quantification capability, certification capability	*Differentiated conformity*: Reconfigures volunteering extracurriculars to develop students’ soft skills and resumes while helping those in need	

*Peripheral separation*: Reconfigure resources to independently address competing prescriptions at the periphery	*Low compatibility*: Forming test-takers (MKT)	*Standardized test preparation extracurricular*: Third-party expertise, third-party course development capability, project management capability, student recruitment capability, classrooms	*Conformity*: Creates a standardized test preparation extracurricular to develop students’ performance at standardized tests	
	*High compatibility*: Forming employable students (MKT)	*Robotics extracurricular:* Third-party expertise, course development capability, network, and product; project management capability; schools’ network; employees’ expertise	*Conformity*: Creates an extracurricular that develop students’ employable skills and resume	
	*Low compatibility*: Forming faithful people (REL)	*Initiation to Christian Life extracurricular*: Religious expertise, religious credentials and reputation, employees’ expertise, schools’ network, physical assets (e.g., church)	*Differentiation*: Reconfigures extracurriculars to reinforce students’ faith	

*Notes:* REL = religious logic; MKT = market logic.

Next, we expand on nested coupling and explain how the four mechanisms operate in practice. For each mechanism, we first define the mechanism; then we explain what led to its adoption and expand on how it was implemented strategically and practically. Throughout, we emphasize dynamics of conformity and differentiation.

#### Managing competing prescriptions within the core

Integrating competing prescriptions within the core can create internal tensions (Besharov and Smith 2014; Gümüsay, Smets, and Morris 2020). To minimize such tensions, the schools privilege the religious logic and subsume or integrate market prescriptions.

We show how the schools adapted their educational philosophy and core technologies around teaching methodologies and related expertise, which are both part of the organizational core (see Hannan and Freeman 1984). We first explain how they adapted their educational philosophy of holistic education to subsume market prescriptions of training students to perform on standardized tests. Second, we describe how they adapted teaching methodologies and related expertise to integrate market prescriptions of self-responsibility and student employability.

Core subsumption refers to the reconfiguration of resources to absorb incompatible market prescriptions into religious ones, which contributes to differentiation while providing some conformity with market prescriptions. By subsuming market prescriptions, organizations minimize potential internal tensions due to incompatibility. We exemplify core subsumption by showing how schools absorb the market prescription of training students to perform on standardized tests into the religious prescription of holistic education. They accomplish this by reconfiguring their educational philosophy.

The marketization of education shifted evaluation criteria toward schools’ positions on national rankings and students’ results on standardized tests (see Table 1). Being ranked became an important source of legitimacy, and a top position in rankings resulted in a source of differentiation for both schools and students. As a result, market-oriented schools concentrated on training students to perform on standardized tests and started “some very aggressive advertising” (Joseph, General Manager, Christ School), “claiming that their admission rates [at universities and based on rankings] are higher” (Father Mark, Top Manager, BJEN). In contrast, the Jesuit perspective on education aligned with the religious logic, where education is holistic; based on a liberal curriculum; and aimed at developing students’ critical thinking skills, connectedness to the self and the community, and humane values (see Table 1). The field-level shift toward school rankings led to this vision losing value: “People assume our quality dropped” (Father Mark). This shift was also problematic because training students solely to perform well on standardized tests was antithetical to the Jesuit educational philosophy. This tension between the two perspectives on education is exemplified in the BJEN’s Educational Common Project (ECP), which establishes guidelines for “reviewing, repositioning, and revitalizing” the organizations (ECP internal document, Foreword). It states:

There is an educational emergency that arises from a market built around education [i.e., marketization]. High competitiveness, driven by the misuse of standardized national and international evaluations, carries the risk of “anthropological reductionism.”…Our way of delivering quality education, however, is not limited to achieving ranking positions. [Our] pedagogical proposal…is centered on the formation of the whole person and for the whole life. (ECP, Assumptions, art. 24–25)

Despite their holistic approach, the schools had historically been committed to academic excellence and, as a result, fared relatively well in rankings, which contributed to their legitimacy. They all ranked among the top ten schools locally (see Table 2). However, fully embracing an educational praxis centered on performance on standardized tests to secure top-ranking spots was a contentious path that entailed sacrificing the core of their educational vision of holistic development. Therefore, rather than pursuing top-ranking positions, the schools decided to differentiate themselves from ranking-oriented competitors by recentering “the purposes, dynamics, actors, conditions, and scope of the educational process” around the principles by which Saint Ignatius lived his life. These became “the inspiration for the educational mission, vision, attitudes, and pedagogical methodology” (internal document, “Conceptual Foundations,” 2018, Apostle School). The goal was to reinforce the holistic aspect of education “based on human and academic excellence…in communion with the educational apostolate of the Society of Jesus.”

The subsumption of market prescriptions within a religious-driven educational philosophy supported a differentiated positioning that still partly conformed to market demands. Rather than pursuing the “unique goal of addressing [standardized] tests and university entrance exams” (Peter, General Manager, Priest School) characteristic of market-oriented competitors, the schools emphasized the cognitive aspects of education as part of a greater whole that also included noncognitive dimensions central to the Jesuit educational philosophy. They did so by reinforcing notions of socioemotional and spiritual development. These were codified in the ECP into a tripartite conceptualization of education composed of “cognitive, socioemotional, and religious-spiritual learning dimensions.” Ivonne (Learning Coordinator, Apostle School) explains this as follows:

Since the ECP, we started developing a series of actions to implement this update of what is called holistic education, making an integral adaptation that looks at the three axes––the cognitive, spiritual-religious, and socioemotional dimensions––in a balanced way, with the same value. The Jesuit schools in general always looked more towards academic performance, while the other two dimensions did not have this kind of pedagogical specificity or intentionality of being clearly aligned, described, and evaluated [as they do now].

This change unfolded in practice through the creation of new course-related resources to train students in socioemotional and spiritual skills. For example, schools translated the tripartite conceptualization of holistic education into learning maps—documents that guide the operationalization of the dimensions across courses, projects, and academic levels. Each of the three dimensions was associated with general competencies and broken down into specific skills and evaluation criteria such as “expected autonomy for each age group” and “respecting different characteristics and points of view” (both for the socioemotional dimension; Kevin, Learning Supervisor, Mary School). These learning maps became key guiding instruments in the transformation of courses and teaching practices, as Ivonne explains:

The teacher can look at the [learning] map, pull the skills related to the age group with which he works…and integrate them in their syllabus…. The teacher can see the relationship between the dimensions and the lesson much better, and the school, too. [After fully implementing it] we will have a curriculum grounded in nine competencies with three competencies for each dimension, and this corresponds to the ECP.

To further support the implementation of the tripartite conceptualization of education, the schools created training sessions and spaces for discussion in which teachers could collaborate to translate the learning maps into teaching material. For example, teachers of the same academic level collaborated to transform both their respective classes and the academic level as a whole. Kevin explains this in the following:

We did an exercise [in teachers’ meetings] to grade on each criterion.…I read [*reading from the learning map*], “Experience school routines with the expected level of autonomy for the age group” and asked, “Let’s go, religion, history, and geography [teachers], how much do you think your [current teaching] strategy addresses this criterion from zero to two?” If a criterion was given a high score, I said, “Great! we’re already achieving this.” Now, there were some, [especially] in the religious-spiritual dimension, that had low scores, therefore, we had to intentionally work on these criteria.…For example, it was very difficult for us to collectively measure this [criterion]: “Values life as sacred in its different manifestations by showing attitudes of respect and care with mediation”.…[Our scores were] almost all zero,…but this was important and led us to rethink our teaching for 2020…and better our teaching activities by discussing how we could come up with a slightly higher score.

By strengthening the alignment of their educational philosophy with the religious logic while absorbing the market prescription, the schools distanced their educational philosophy from that of their market-oriented competitors, which led to a unique competitive positioning that balanced pressures for conformity and differentiation. This differentiated positioning is widely acknowledged by parents, as the following parent’s review of the Priest School’s strengths shows:

Here, there is this idea of human development. They learn to care about the other. It is not like in other schools, which are very focused on students’ performance, grades, [and] ENEM placement. We know that all schools work towards this.…But we have this perception that here, we also have something very nice in terms of social and human development.

We next turn to the second mechanism through which organizations integrate compatible prescriptions within the core, which provides greater conformity with market prescriptions while preserving some differentiating elements.

Core integration refers to the reconfiguration of resources to blend compatible market prescriptions with religious ones, which modernizes preexisting resources while capitalizing on the opportunities they afford for differentiation. Compared with subsumption, prescriptions can be blended without risking high internal tensions due to their compatibility. We exemplify core integration by showing how schools integrate the market prescription of forming self-responsible and entrepreneurial students with the religious prescription of Jesuit autonomy. They accomplish this by reconfiguring core technologies associated with teaching methods and related expertise.

Marketization brought forth the precept of self-responsibility, characterized in education by forming entrepreneurial and self-regulated learners (see Table 1). Typically, self-responsible entrepreneurship serves neoliberal ideals. In education, forming students as self-responsible entrepreneurs equips them with qualities needed in the contemporary job market, such as ambition, drive, self-motivation, and self-reliance (Davies and Bansel 2007). An example of how this affected educational praxis is reflected in market-oriented schools’ transition from a lecture-based curriculum created by teachers and pedagogic coordinators to a student-centered curriculum oriented around active learning methodologies to form self-responsible and entrepreneurial learners (Roderick 2019).

This student-centered approach to learning differed from the teacher-led lectures that defined the Jesuit teaching methodology. BJEN schools recognized the need to adapt to “a teaching approach where the student is more active [and that] is trending now [in the private sector]. There is this inverted classroom thing, for example, where the teacher gives the students’ part of the responsibility to learn” (Marcio, Expert, Apostle School). The schools also faced increased pressure from parents and students to change their teaching methods “to keep up with this trend…through other ways of teaching” (Anelise, Academic Director, Christ School).

Perhaps surprisingly, given the theonomy and hierarchical characteristics of organized religions, the market prescription of forming self-responsible and entrepreneurial students was compatible with the religious prescription of self-autonomy to serve God. We exemplify the latter with an excerpt from “Ignatian Pedagogy: A Practical Approach” (International Commission on the Apostolate of Jesuit Education 1993 **)**, “one of two key documents which emerged from a lengthy and worldwide reflection on Jesuit education in the 1970/80s” (http://jesuitinstitute.org/Pages/IgnatianPedagogy.htm):

The ultimate goal of Jesuit education is…action…suffused with the spirit and presence of Jesus Christ [that] urges students to *self-discipline and initiative* (art. 12) ACTION: [education should] *enable the [student] to know the will of God and to do it freely*…[students should] make decisions in a wide variety of situations about what actions were to be done…[to] contribute intelligently and effectively to the welfare of society (art. 59)…*meanings, attitudes, values which have been interiorized, made part of the person, impel the student to act*…consistent with this new conviction (art. 62). [The goal is to form a student] who *becomes self-motivated by his or her own integrity and humanity to make conscious, responsible choices* (art. 70). (International Commission on the Apostolate of Jesuit Education 1993, emphases added)

The schools integrated the market prescription by blending neoliberal self-responsibility with Jesuit autonomy, creating a Jesuit version of a self-responsible entrepreneurial student: self-responsibility at the service of religious ideals. For example, the ECP states that the schools should “form global citizens who are leaders in service; academic; competent and ethically responsible; and committed to building a more just, fraternal, supportive and inclusive society” (Introduction).

The schools accomplished such integration by moving “from a more knowledge-based curriculum to one in which the learning center is the student” (James, Pedagogic Coordinator, Apostle School). To do so, they reconfigured teaching methodologies and related skills to incorporate “active methodologies [that] make students participate effectively and affectively in their educational process…[and] realize the level of responsibility that they have over this process” (Kevin, Learning Supervisor, Mary School). Rather than passively listening to class lectures, students became actively involved in the classroom experience using new methods such as the reverse classroom technique and project-based learning with the intent of “developing the skills and competencies necessary to exercise autonomy” (ECP, art. 42).

To support teachers through this transition, the schools “invested in teacher training in new methodologies to train [teachers] from different classes in different methodologies in which the student is the protagonist” (Carolina, Pedagogic Coordinator, Christ School). These training sessions transformed teaching activities. For example, Lia, a Portuguese teacher at Christ School, created an entire learning unit for fifth-graders using active learning methodologies. She exemplifies here both the use of active learning by her students and the integration of market prescriptions with Jesuit values of inclusivity and compassion:

Their textbook has some comic strips…[telling a story of] a character who suffers bullying. I thought to approach this issue in a way that is not as superficial and that resonates with them…So, I proposed a debate and asked them to prepare something at home. [The next day,] I posed a [debate] question…and small groups of students…debated among themselves with what they had brought from home from previous conversations they had had with their families…I was just the organizer. I avoided talking and let them hear what they were saying…they got involved, and they felt part of the process.…[this is in line with what] I see in the ECP when it talks about the student as a protagonist…this is knowledge that goes beyond content…it is about action [and] reflecting on [the impact of] one’s actions on others.

The integration of active learning methodologies helped the schools in conforming to the market prescription of forming self-responsible and entrepreneurial students, redressing their legitimacy. For example, in comparing Priest School with one of its elite competitors, one mother exemplifies the success of the new approach in helping her children develop self-accountability:

This is similar to what we had [in the previous market-oriented school]. By the way, it is one of the other reasons why I transferred them to Priest School. They gained an immense sense of responsibility here. Today, I see the difference in my two children.…[They] learned to be responsible.…I don’t even keep track of their homework because they have gained so much autonomy.

However, the schools embedded the market prescription into religious ideals, which supported their differentiation. Anna (Pedagogic Coordinator, Christ School) emphasizes this idea by explaining the kind of entrepreneurial behavior students adopted through their participation in active learning methodologies:

We are working with this idea where [students] take the initiative [to save the environment] themselves so that they do good not only for themselves but for others too.…So, we work with [active learning] projects [on environmental awareness].…So, I can’t say to a student that it is necessary to take care of [the environment], turn off a lamp, or throw waste in the garbage. [Rather,] we need to *propose* this change.…[We knew this worked] when the fathers [of our students] came here and said, “Look, teacher, my daughter went to the mall today, saw a plastic bottle on the floor and said: ‘Daddy, this can’t be on the floor, this has to be in the plastic basket!’” These are small things that we see in our students; the changes are happening.

In summary, we explain in this section how organizations reconfigure their resources to manage competing prescriptions within the core. We presented two mechanisms that vary depending on whether prescriptions are compatible. When faced with incompatible market prescriptions, organizations absorb them into religious ones, which mostly differentiates the schools from market-oriented competitors. When faced with compatible market prescriptions, they blend them with religious prescriptions, which heightens conformity but also supports differentiation. We next turn our attention to how schools manage competing prescriptions at the periphery.

#### Managing competing prescriptions at the periphery

In contrast to managing competing prescriptions within the core, doing so at the periphery is less likely to cause tensions, providing organizations with greater latitude for strategic action (Besharov and Smith 2014; Gümüsay, Smets, and Morris 2020). At the periphery, organizations integrate market prescriptions and address religious and market prescriptions separately.

We illustrate how the organizations manage prescriptions at the periphery by transforming extracurriculars. Extracurriculars are peripheral because they are additional learning activities that complement the core. They have little impact on educational philosophy or core technologies. We first briefly discuss the integration of prescriptions at the periphery. We then explain how a greater range of possible strategic responses allows the schools to respond to competing prescriptions separately and address them fully.

Peripheral integration refers to the reconfiguration of resources to blend compatible market and religious prescriptions. Because this mechanism mirrors core integration (see Table 3), we evidence it in Web Appendix B to concentrate on the last mechanism, which is theoretically different from the first two.

Peripheral separation refers to the reconfiguration of resources to independently address competing prescriptions in ways that fully correspond to religious or market demands. We exemplify peripheral separation by showing how schools directly respond to market or religious prescriptions by transforming extracurriculars. They accomplish this by adapting and developing resources and bundling them with those of third parties to strengthen responses to market prescriptions and by leveraging existing resources to reinforce responses to religious prescriptions. This mechanism alleviates the limitations of responses within the core.

First, the schools developed extracurriculars to respond to incompatible prescriptions not fully addressed previously. For example, for core subsumption, schools absorbed the market prescription of training students to perform on standardized tests because it conflicted with their holistic educational philosophy. However, the schools could “not deny that families and students also want to pass university entrance exams…So, we are attentive to this, too; we are not pretending that we don’t have anything to do with that” (Peter, General Manager, Priest School).

Rather than recentering their educational philosophy around preparing students for standardized tests and risking high internal tensions, the schools addressed this market prescription fully by addressing it at the periphery. They did so by combining the resources of third parties with their own. To manage this combination, some schools assigned new responsibilities to members of their pedagogical teams, and others created a new position. Their work led to the joint creation by schools and third parties of an extracurricular standardized test preparation program. Schools varied in how they implemented it, but it usually involved lectures and workshops from guest speakers on how to prepare for the tests, the administration of test simulations, and follow-up sessions on simulation results to identify “the disciplines in which students did not perform as well” (Danton, Academic Director, Apostle School). The simulations were sourced from third parties, which also provided detailed performance reports for the students and schools. “The majority of the schools in the network” used the same [simulation] provider (Guillaume, Academic Director, Mary School), and they combined and coordinated their national reach with that of the third party so that “students [would] do the simulation at the same time as other students from various regions, allowing for a better appreciation of their performance…and reflecting the reality they would experience [when taking the ENEM test]” (ENEM Simulation pamphlet, Jesus School).

Responding to market prescriptions at the periphery provides more latitude for strategic responses. The schools used this greater realm of action to address the market prescription of training students for standardized tests directly while maintaining a distance between this activity and their core educational offer. For example, rather than developing this activity themselves, as in core subsumption and core integration, they outsourced the development to a third party. The schools also limited training to coordinators responsible for extracurricular activities (vs. systemically training teachers), and they scheduled these activities on the weekend (outside of regular course hours). These distancing strategies would have been impossible within the core. Managing prescriptions at the periphery allowed the schools to better conform to this important market prescription, contributing to their legitimacy while insulating their market-oriented response from their core educational offerings, which minimized potential internal tensions.

Second, the schools diversified extracurriculars to complement their response to prescriptions already addressed within the core. For example, the schools strengthened their response to the market prescription of improving students’ employable skills by developing extracurriculars that “families indicated they wanted more of at school” (Marta, Extracurricular activities coordinator, Apostle School). These included the “mini-enterprise” project developed with the local branch of the local Youth Achievement Group, which expands students’ “practical experience in economics and business” (mini-enterprise pamphlet, Apostle School) and the “robotics project” developed with “educators…trained in control and automation” (robotics pamphlet, Mary School).

As with the standardized test preparation extracurricular program, the schools either created new positions or assigned new responsibilities to existing teams to manage these new extracurriculars and relationships with third parties recruited to support their development. For example, to develop the robotics project, Mary School partnered with a company specialized in this area. The “whole service is outsourced, and no educators from the school are responsible for teaching this project” (Guillaume, Principal). The extracurricular program “provides experience with using technology as a tool to solve problems and challenges that we encounter in various real-life contexts…stimulating sociability; communication and the development of skills such as cooperation, pro-activeness, conflict management, commitment, being result-focused and logical thinking” (robotics pamphlet, Mary School), all of which are qualities demanded by employers (Illouz 2007). This also enabled the schools to mimic the offerings of their competitors, which increasingly invested in robotics. The schools bundled their resources, such as classroom space and student recruitment capabilities, with third-party resources, such as course development capabilities where “course material necessary for the classes was the responsibility of the third party” (Guillaume).

The management of market prescriptions at the periphery allows the schools to “boldly implement [market-oriented] optional activities” (Anelise, Academic Director, Priest School) and address them directly by, for instance, training students in test-taking and developing students’ employable skills (see Table 1). This contributes to the schools’ conformity. They also manage prescriptions on the periphery to differentiate themselves, which we describe next.

Third, the schools transformed extracurriculars to reinforce their responses to religious prescriptions. They did so in part because the reconfiguration of resources within the core to integrate market prescriptions minimized the religious aspects of some courses. For example, the schools adapted how religion was taught in their educational program by blending religious and market prescriptions to address a “movement of laicization…[as] a reality that is changing…[and] less focused on the Church” (Aria, History Teacher, Christ School). Rather than teaching religion, they created a course on “Human and Christian Formation” that emphasized “human rights…[and was] less focused on the Church [and more] on issues such as bullying, prejudice, more general things” (Aria). Or, put differently, the integration of the market prescription of developing students’ emotional intelligence resulted in a lesser focus on teaching the Bible.

In response, the schools adapted extracurricular pastoral activities “to rescue the Jesuit identity at the schools” (Danton) by capitalizing on their preexisting resources such as knowledge of religious practices, employees who are members of the clergy, and religious assets (e.g., a church on school grounds). For example, they created “weekend retreats for…young people wishing to deepen their spirituality [and] a day of prayer and exercises to learn about the methodology of Ignatian spirituality” (Danton). They also expanded the extracurricular activity “Catechesis” into a five-year “Initiation to Christian Life” program with the objectives of “awakening in children and families the desire to know more about and meet Jesus Christ [and] to live the faith in an authentic and true way” (extracurricular activities pamphlet, Christ School). Managing religious prescriptions in the periphery allowed the schools to concentrate on “everything that is biblical teaching” (Aria) while isolating this highly religious activity from their core educational offer. These religious activities differentiate the schools from market-oriented competitors and address the needs of parents and students who have not yet embraced market-driven choice criteria as well as those who want both a religious and market-driven school. Aria expands: “The families still believe in Christian values…[as] a reference for them as family and society.…They see it as a way to give the kids a [religious] direction beyond what the school offers in the core curriculum.”

In summary, we explain in this section how organizations reconfigure their resources to manage competing prescriptions at the periphery by separating or integrating them. On the one hand, they separate incompatible prescriptions that cannot be addressed in the organizational core due to high risks of internal tensions and strengthen responses to compatible ones. They also use peripheral separation to reinforce responses to religious prescriptions. On the other hand, they blend compatible prescriptions through a process akin to core integration. When combined with core subsumption and core integration, managing prescriptions at the periphery balances pressures for conformity and differentiation.

Finally, previous work on hybridization shows heterogeneous responses to complexity (Greenwood et al. 2011). Rather, the schools we examined have transformed in relative uniformity. We explain this discrepancy in Web Appendix C. The uniformity of the implementation of nested coupling can be attributed to the strategic role of the BJEN at the network level, providing tools and resources to facilitate implementation within schools and monitoring the progress of each school. Next, we expand on factors explaining heterogeneous responses and conclude by describing our contributions, providing managerial recommendations, and expanding on avenues for future research.

## Discussion

Marketization shifts the dominant institutional logic of a field to the market logic, which creates a unique set of challenges for established organizations previously underexamined in extant research. First, it opens a field to market-style competition mechanisms and consumer choice, which undermines the legitimacy of established organizations. Second, the market logic is typically in conflict with those that preexisted marketization, which creates a potential for internal conflicts in those established organizations wishing to embrace it. To respond to marketization, organizations need to balance demands for conformity and differentiation.

We introduce nested coupling as a strategic response to marketization. Nested coupling creates a hierarchical blending of preexisting and market logics where the preexisting logic informs the integration of the market logic. The integration of the market logic can lead to internal conflicts, which organizations manage by integrating market prescriptions differently between core and peripheral resources. Our findings show how organizations prioritize the religious logic within the core, which minimizes risks for internal tensions. They do so by subsuming incompatible market prescriptions into religious ones and by integrating compatible marketing prescriptions. At the periphery, organizations have greater strategic latitude to engage more directly with market prescriptions and address limitations of responses within their core. They use this latitude to respond to market or religious prescriptions separately to support either conformity or differentiation. They also blend compatible prescriptions. In summary, nested coupling allows firms to conform to competing logics while differentiating themselves from competitors. We provide speculative examples of the application of nested coupling to the museum and health care fields in Web Appendix D.

Our contributions are twofold. First, we introduce nested coupling as a novel hybridizing strategy and show how it provides a strategic avenue for established organizations to respond to marketization. We contribute to existing work on hybridity by expanding beyond conformity and demonstrating how hybridization also supports differentiation. Apart from a few exceptions (Dalpiaz, Rindova, and Ravasi 2016; Ertimur and Coskuner-Balli 2015), previous work has almost exclusively discussed how hybridization helps firms conform to competing institutional logics (Greenwood et al. 2011). It also has not examined how organizations balance demands for conformity and differentiation. Moreover, existing work concentrates on explaining hybridization through the separation or integration of logics as a whole (see Battilana, Besharov, and Mitzinneck 2017). In contrast, we introduce four mechanisms that operate at the prescription level rather than the logic level. This is an important theoretical insight that informs how organizations can separate and integrate prescriptions from competing institutional logics in a complementary fashion both within the core and at the periphery. Last, to our knowledge, our model represents the first empirically grounded effort to integrate logics with resources. This theoretical combination offers a new lens to study organizations and marketing strategy.

Second, in our discussion, we leverage nested coupling to provide theoretical insights into strategic orientation. More specifically, we expand on the idea previously introduced that work on strategic orientation assumes that the market logic drives a field. In contrast, given that many fields are not solely governed by the market logic, we propose that the dominant logic of a field affects the implementation of a strategic orientation. We exemplify this idea by showing how market driving operates differently under market versus nonmarket logics. In addition, nested coupling informs how organizations can adopt hybrid strategic orientations. Continuing with the example of market driving, we show that nested coupling provides a theoretical mechanism to understand a hybrid driving–driven strategic orientation. Next, we discuss market factors that lead firms to respond to marketization differently.

### Heterogeneous Adaptations to Marketization

Nested coupling offers a strategy for established organizations to adapt to marketization. We expand on nested coupling to explain heterogeneity in patterns of adaptations. We do so by combining the four mechanisms of nested coupling differently. Two factors explain heterogeneity: how insulated an established organization is from the market logic and how dominant the market logic is in a field.

First, the degree to which organizations are insulated from market-style mechanisms of consumer choice and competition determines the potential pressure exerted by the market logic on financial resources. Organizations in marketizing fields typically have four sources of financing: patrons, governments, endowments, and customers (Bell 2016). Some organizations are highly insulated from the market, such as privately funded collections open to the public (e.g., the Menil Collection) and organizations catering to highly traditional populations (e.g., yeshivas). Their revenues are less dependent on the market, or their customers are less likely to shift to market-based choice criteria. Thus, they are less pressured to respond to market prescriptions. In cases where organizations are highly insulated from the market logic, we expect little adaptation. It is possible that organizations adapt at the periphery, whereas there is little need to adapt within the core. Insulated organizations maintain their alignment with a preexisting logic. We term this pattern a traditional orientation.

Most organizations are less insulated from the market, such as government-funded museums or organizations that cater to a modern population that increasingly eschews traditions (e.g., the organizations we studied). Their sources of revenue are likely to be influenced by marketization. For example, since the 1970s, the market logic has expanded its reach, and governments have defunded nonprofits, education, and health care (Baltodano 2012; Eräranta, Moisander, and Penttilä 2020; Harvey 2005). As a result, the revenues of these organizations have increasingly depended on adapting to market-style mechanisms of consumer choice and competition.

Our second condition, the dominance of the market logic in a field, is thus of importance. In a less marketized field, such as the one in which the organizations we examined evolved at the end of the 1990s, the market logic is peripheral to a preexisting logic. Consumers have yet to become a dominant source of legitimacy or embrace market prescriptions. Established organizations can thus draw from the authority afforded by a preexisting logic and are likely to maintain a traditional orientation. They might slowly adapt at the periphery to accommodate the rise of the market logic. For example, some of the schools we studied started to respond to market prescriptions at the periphery by offering market-oriented extracurriculars in the late 1990s, such as Apostle School, which launched the “mini-enterprise” extracurricular program. They had yet, though, to adapt within the core.

However, the market logic is typically seen as hegemonic (Harvey 2005). It is thus likely that its influence will increase over time. Therefore, less insulated organizations will face increased pressures to adapt to market prescriptions.

As marketization intensifies, the market logic gains dominance in the field and competes with preexisting logics. This is the market environment we studied. At this stage, established organizations can opt to practice nested coupling to adapt to marketization and maintain their competitiveness. We qualify this positioning as a nested traditional orientation. We hypothesize this to be the most productive strategic avenue, as organizations that continue with a traditional orientation will probably experience declining market share.

Finally, as exemplified by many fields in the United States where marketization has run its course, the market logic will likely supplant preexisting logics over time. In these fields, preexisting logics are still present, but they evolve in the periphery of the market logic. We see two possibilities for established organizations in such cases. First, they can opt for a traditional or a nested traditional orientation and operate on the market periphery, embracing a niche positioning strategy. Second, they can further hybridize to become more market oriented. In such cases, we expect organizations to nest the preexisting logic within the market logic to maintain some differentiation from competitors, inverting the relationship between preexisting and market logics presented in core subsumption. This inversion in how logics are nested should allow firms to maintain their differentiation from other firms that solely engage with the market logic while conforming to increasingly authoritative market prescriptions. In addition, we hypothesize that responses initially made at the periphery of the organization will move to the core. This last stage, which we qualify as a nested market orientation, might be the endpoint of the strategic adaptation of established organizations in a marketized field. Exactly how this shift happens over time is an avenue for future research.

### How Institutional Logics Inform Strategic Orientations

Shifts in institutional logics are frequent and not only driven by marketization. For example, new logics have transformed many fields over the last century, such as the therapeutic logic, which changed management (Illouz 2007); the craft logic, which transformed the beer and coffee markets (Heying 2010); and the sustainability logic, which reshaped many fields (Bothello and Salles-Djelic 2018).

Our theoretical insights can help navigate shifts in logics in at least two ways. First, a logics perspective can help firms evaluate how to deploy a specific strategic orientation. Second, instead of embracing a strategic orientation as a whole (e.g., Gebhardt, Carpenter, and Sherry 2006) or refusing to do so (Press et al. 2014), nested coupling suggests that firms can adopt hybrid strategic orientations. We expand on these ideas by extending our theoretical insights to market-driven and market-driving orientations (Jaworski, Kohli, and Sajay 2000).

First, a logics perspective can help firms evaluate how to deploy a strategic orientation. For example, market driving is conceptualized as a strategic orientation in which firms strive to change the market structure or the behavior of competitors or consumers (Jaworski, Kohli, and Sajay 2000). Market driving capitalizes on market-style competitive strategies, such as buzz marketing, disruptive innovations, or status games (Humphreys and Carpenter 2018).

However, our historical overview of the Brazilian education market points to another, previously unexplored, form of market driving. In nonmarketized fields, firms drive markets not through innovation, marketing, or status, but rather by using the authority and legitimacy afforded by a particular logic. Prior to marketization, hospitals, museums, and schools mostly drove markets because the dominant logic afforded authority and legitimacy to professionals.

Thus, market driving operates differently depending on the logic that governs a field. In a field dominated by the market logic, market driving capitalizes on market principles to transform a market. In nonmarketized fields, it concentrates on a source of authority and legitimacy unrelated to market principles. In short, logics can inform the execution of a strategic orientation.

Second, nested coupling can inform the adoption of hybrid strategic orientations. In our findings, the established organizations responded to marketization by becoming at least partially market driven. They did so by nesting market prescriptions into religious ones. We use this insight to explain how firms can be both market driving and market driven (see Jaworski, Kohli, and Sajay 2000)—a driving–driven hybrid—and extend previous work that concentrated solely on a market-driving orientation (e.g., Humphreys and Carpenter 2018; Maciel and Fischer 2020).

Historically, the religious logic provided the authority and legitimacy required for religious schools to drive the market. When faced with marketization, the schools maintained their alignment with the religious logic and capitalized on their legitimacy as religious and educational authorities to reassert the value of a Jesuit education. They, at least partly, strove to continue driving the market. However, by transforming some of their resources to align with market demands, they also addressed customer needs and responded to increased competitive pressures. That is, they became at least partly market driven. Nested coupling thus created a hybrid driven-driving orientation. We hypothesize that the balance between conformity and differentiation created by nested coupling might benefit market-driving efforts by providing authority and legitimacy under different logics. For example, by conforming to at least some market demands, a firm can derive legitimacy from consumers. By embracing a nonmarket logic, it can also draw from another source of legitimacy to support market-driving efforts.

A logics perspective also offers an alternative route to practice market driving. Rather than changing consumers’ preferences and competitors directly, firms can strive to introduce or maintain a logic that fits their organizational actions (e.g., Ertimur and Coskuner-Balli 2015). For example, from a logics perspective, craft breweries could strive to influence consumer preferences by introducing craft prescriptions to the market, such as a passion for quality, in addition to continuously innovating and building market reputation (cf. Maciel and Fischer 2020). This approach illustrates a mesolevel cultural shaping strategy rather than microlevel ones such as shaping “the choice sets, criteria, and benefit packages that customers buy” (Jaworski, Kohli, and Sahay 2000, p. 45). A logics perspective on market driving could also shed light on a wider range of market elements that can be shaped by firms, from consumer preferences and competitor behavior to the value of professional identities and organizational forms to the types of relationships possible between firms and consumers.

To conclude, a logic perspective informs not only how established organizations can adapt to marketization but also how to execute or hybridize strategic orientations. We encourage the further advancement of this research program. Given the wealth of research on the internal management of competing logics (see Battiliana, Besharov, and Mitzinneck 2017; Greenwood et al. 2011), we suspect that we have only scratched the surface of how logics can benefit the competitiveness of firms.

## Supplemental Material

Supplemental Material, sj-pdf-1-mrj-10.1177_0022243721999042 - How Established Organizations Combine Logics to Reconfigure Resources and Adapt to Marketization: A Case Study of Brazilian Religious Schools Supplemental Material, sj-pdf-1-mrj-10.1177_0022243721999042 for How Established Organizations Combine Logics to Reconfigure Resources and Adapt to Marketization: A Case Study of Brazilian Religious Schools by Pierre-Yann Dolbec, Rodrigo B. Castilhos, Marcelo J. Fonseca and Guilherme Trez in Journal of Marketing Research
