# Supplementary material for: How Established Organizations Combine Logics to Reconfigure Resources and Adapt to Marketization: A Case Study of Brazilian Religious Schools
Source: J Mark Res. 2021 Jul 13;59(1):118–35. doi: 10.1177/0022243721999042 (PMC13038147; doi:10.1177/0022243721999042)
Supplement: Supplemental Material, sj-pdf-1-mrj-10.1177_0022243721999042 - How Established Organizations Combine Logics to Reconfigure Resources and Adapt to Marketization: A Case Study of Brazilian Religious Schools [file sj-pdf-1-mrj-10.1177_0022243721999042.pdf]

HOW ESTABLISHED ORGANIZATIONS COMBINE LOGICS TO RECONFIGURE RESOURCES AND ADAPT TO MARKETIZATION: A CASE STUDY ON BRAZILIAN RELIGIOUS SCHOOLS

Web Appendix A: References for Ideo-typical Logics

Akkari, Abdeljalil (2013), "Blurring the Boundaries of Public and Private Education in Brazil," *Journal of International Education and Leadership*, 3 (1), 1–13.

Anderson, Gary (2017), "Participatory Action Research (PAR) as Democratic Disruption: New Public Management and Educational Research in Schools and Universities," *International Journal of Qualitative Studies in Education*, 30 (5), 432–49.

Apple, Michael W. (2006), "Understanding and Interrupting Neoliberalism and Neoconservatism in Education," *Pedagogies: An International Journal*, 1 (1), 21–26.

Attick, Dennis (2017), "Homo Economicus at School: Neoliberal Education and Teacher as Economic Being," *Educational Studies*, 53 (1), 37–48.

Buchanan, Michael T. and Adrian-Mario Gellel (Eds.) (2019), *Global Perspectives on Catholic Religious Education in Schools: Volume II: Learning and Leading in a Pluralist World*, Singapore: Springer.

Basu, Ranu (2004), "The Rationalization of Neoliberalism in Ontario's Public Education System, 1995–2000," *Geoforum*, 35 (5), 621–34.

Brazil Casa Civil (1996), "Lei nº 9.394", available at http://www.planalto.gov.br/ccivil_03/LEIS/L9394.htm.

Brazil Casa Civil (1988), "Constituição da República Federativa do Brasil", available at http://www.planalto.gov.br/ccivil_03/constituicao/constituicao.htm.

Burke, Kevin (2012), "Strange bedfellows: The New Neoliberalism of Catholic Schooling in the United States," *Journal of Pedagogy*, 3 (2), 177–97.

Butts, R.Freeman (1978) *Public Education in the United States: From Revolution to Reform*. Holt, Rinehart and Winston.

Carbonari, Pâmela (2015), "Tabuada, Lousa — e Lucro," *Revista Exame*, available at: https://exame.abril.com.br/revista-exame/tabuada-lousa-e-lucro/.

Clayton, Matthew and David Stevens (2018), "What is the Point of Religious Education?," *Theory and Research in Education*, 16 (1), 65–81.

Collins, A. & Halverson, R. (2009). *Rethinking Education in the Age of Technology: The Digital Revolution and the Schools.* New York: Teachers College Press.

Connell, Raewyn (2013), "The Neoliberal Cascade and Education: An Essay on the Market Agenda and its Consequences," *Critical Studies in Education*, 54 (2), 99–112.

Curi, Andréa Zaitune, and Naércio Acquino Menezes-Filho (2009), "A Relação Entre Educação Pré-primária, Salários, Escolaridade e Proficiência Escolar no Brasil," *Estudos Econômicos*, 39 (4), 811–50.

Diamond, John B., Antonia Randolph, and James P. Spillane (2004), “Teachers’ Expectations and Sense of Responsibility for Student Learning: The Importance of Race, Class, and Organizational Habitus,” *Anthropology & Education Quarterly,* 35 (1), 75–98.

Evans, David and Tobin Miller Shearer (2017), “A Principled Pedagogy for Religious Educators,” *Religious Education*, 112 (1), 7–18.

Fuchs, John Andreas (2017), “‘It Takes a Village’ – (Catholic) Education in the 21st Century,” in *Schools in Transition*, P. Siljander, K. Kontio, and E. Pikkarainen, eds., Rotterdam: Sense Publishers, 229–39.

Giroux, Henry (2002), “Neoliberalism, Corporate Culture, and the Promise of Higher Education: The University as a Democratic Public Sphere,” *Harvard Educational Review,* 72 (4), 425–64.

Grace, Gerald and Joseph O’Keefe (Eds.) (2007). *International Handbook of Catholic Education: Challenges for School Systems in the 21st Century*. Dordrecht: Springer.

Groome, Thomas H. (2005), “What Makes a School Catholic?,” In *The Contemporary Catholic School: Context, Identity and Diversity*, eds. Terence H. McLaughlin, S.J. Joseph O’Keefe, and Bernadette O’Keeffe, 106–124. London: The Falmer Press.

Jesuit Institute (n.d.), “Ignatian Pedagogy Document,” *Jesuit Institute London,* http://jesuitinstitute.org/Pages/IgnatianPedagogy.htm.

Johnson, James, Diann Musial, Gene E. Hall, Donna M. Gollnick, and Victor L. Dupuis (2004). *Introduction to the Foundations of American Education,* 13th Edition. Allyn and Bacon.

Katz, Michael B. (1976), “The Origins of Public Education: A Reassessment,” *History of Education Quarterly*, 16 (4), 381–407.

Klein, Pe. Luiz Fernando (2016), “Trajetória Da Educação Jesuítica no Brasil,” presented at the *Ciclo de Debates*, May 21st, Pateo de Collegio, 1–21.

Kontio, Kimmo, Eetu Pikkarainen, and Pauli Siljander (2016). *Schools in Transition: Linking Past, Present, and Future in Educational Practice.* Boston, MA: Sense Publishing.

Lakes, Richard D. and Patricia A. Carter (2011), “Neoliberalism and Education: An Introduction,” *Educational Studies*, 47 (2), 107–10.

Libâneo, José Carlos (2016), “Políticas Educacionais No Brasil: Desfiguramento da Escola e do Conhecimento Escolar,” *Cadernos de Pesquisa*, 46 (159), 38–62.

Lingard, Bob (2010), “Policy Borrowing, Policy Learning: Testing Times in Australian Schooling,” *Critical Studies in Education*, 51 (2), 129–47.

Maciel, Lizete Shizue B., and Alexandre Shigunov Neto (2006), “Brazilian Education in the Pombaline Period: A Historical Analysis of the Pombaline Teaching Reforms,” *Educação e Pesquisa*, 32 (3), 465–76.

Mockler, Nicole (2018), “Early Career Teachers in Australia: A Critical Policy Historiography,” *Journal of Education Policy*, 33 (2), 262–78.

Nasaw, David (1979), *Schooled to Order: A Social History of Public Schooling in the United States,* New York: Oxford University Press.

Nieto, Sonia (2005), "Public Education in the Twentieth Century and Beyond: High Hopes, Broken Promises, and an Uncertain Future," *Harvard Educational Review*, 75 (1), 43–64.

Nurwanto and Carole M. Cusack (2017), "Addressing Multicultural Societies: Lessons from Religious Education curriculum policy in Indonesia and England," *Journal of Religious Education*, 64 (3), 157–78.

O'Keefe, Joseph, Joseph O'Keefe, and Bernadette O'Keefe (2003), *The Contemporary Catholic School: Context, Identity and Diversity*. Washington: The Falmer Press.

Olssen, Mark and Michael A. Peters (2005), "Neoliberalism, Higher Education and the Knowledge Economy: From the Free Market to Knowledge Capitalism," *Journal of Education Policy*, 20 (3), 313–45.

Posamentier, Jordan, Robin Lake, and Paul Hill (2017), "How States Can Promote Local Innovation, Options, and Problem-Solving in Public Education," *CRPE Reinventing Education.*

Rasmussen, Bruna (2019), "Educação Fora da Caixa: Conheça Escolas Onde o Aprendizado vai Muito Além da Lousa e do Caderno," *Hypeness*, available at: https://bit.ly/3aZZ7ke.

Ravitch, Diane and Lois A. Stoehr (2017), "The Death and Life of the Great American School System," in *Early Learning*, Sharon E. Shaffer, ed., Routledge, 125–28.

Salvador, Evilasio, Natalia de Souza Duarte, Samuel Pantoja Lima, and Luiza Andrea Mangabeira da Costa (2017), "Privatização e Mercantilização da Educação Básica no Brasil," *Confederação Nacional dos Trabalhadores em Educação*.

Senefonte, Fábio Henrique Rosa (2018), "The relationship between religion and education in Brazil," *Revista Linhas*, 19 (40), 434–54.

Shore, Cris (2010), "Beyond the Multiversity: Neoliberalism and the Rise of the Schizophrenic University," *Social Anthropology*, 18 (1), 15–29.

Souza, S.A. (2012), "A Introdução do Empreendedorismo na Educação Brasileira: Primeiras Considerações," *Educação & Linguagem*, 15 (26), 77–94.

Stacey, Meghan (2017), "The Teacher 'Problem': An Analysis of the NSW Education Policy 'Great Teaching, Inspired Learning,'" *Discourse: Studies in the Cultural Politics of Education*, 38 (5), 782–93.

Thomas, Janet Y. and Kevin P. Brady (2005), "The Elementary and Secondary Education Act at 40: Equity, Accountability, and the Evolving Federal Role in Public Education," *Review of Research in Education*, 29 (1), 51–67.

Web Appendix B: Peripheral Integration

Peripheral integration. Peripheral integration refers to the reconfiguration of resources to blend compatible market prescriptions with religious ones, which modernizes pre-existing resources while capitalizing on the opportunities they afford for differentiation. In comparison to core integration, peripheral integration occurs on the organizational periphery. We exemplify peripheral integration by showing how schools integrate the market prescription of contributing to students' competitiveness for university admission and on the job market with the religious prescription of forming faithful and fraternal people. They accomplish this by transforming extracurriculars associated with volunteering activities.

Before marketization, the schools offered an array of volunteering opportunities for students to help those in need. These included 'social campus trips' to religious missions and local communitarian actions, which initially answered the religious prescription of forming fraternal and good people by teaching "love and charity … to awake students to a faith that … bring them closer to the reality of those in need" (Volunteering activities webpage, *Christ School*). However, with marketization, extracurriculars solely oriented at helping the disadvantaged became "much questioned by the parents about what it means [in terms of the development of the students]" (Danton Academic Director, Apostle School). Claudio (Volunteering Coordinator, *Apostle School*) adds that "[Volunteering was seen] as a Church thing … And we saw the demand of many of our students who want to go in exchange [programs] in the United States or Canada. Many universities want this [a student resume that has meaningful extracurricular experiences] … So, we made this decision to reposition our volunteering [extracurriculars]".

The schools responded by reconfiguring volunteering extracurriculars to strengthen how they addressed market prescriptions of students' future employability and competitiveness for university admission (see Table 1). They did so by changing volunteering extracurriculars to emphasize the development of soft skills such as "leadership, teamwork, and self-knowledge" (Claudio). The schools also transformed the activities to support the building of students' experiential resumes by providing volunteering certificates. Students' soft skills and experiential resumes are essential tools for admission at prestigious universities and future employment (Rivera 2015). Transforming volunteering extracurriculars helped the schools address market prescriptions.

To support the transformation of volunteering extracurriculars, the schools reconfigured their resources. For example, they developed resources to quantify students' involvement in volunteering extracurriculars to support students' efforts to build their resume. The schools developed "a system … to have greater control on attendance" and track students' participation in volunteering extracurriculars in a way that is "much more organized" by "registering them [to] count their volunteering hours" (Claudio). Claudio explains

> [For this volunteering activity], we take attendance. Every time students participate, they mark being present or not. At the end of the semester, we count the number of hours and register them in the system. This creates a record of students' participation [to volunteering activities] to grant them a certification. There are [also] other sources of volunteering hours that are included. The student is also asked to think of a goal [of volunteering hours] to achieve.

The quantification of self, such as the quantification of student productivity, is a well-acknowledged response to the demands of the market logic to address self-responsibility (e.g., Moore and Robinson 2016). Awarding volunteering certifications also addresses market prescriptions. Yet, the volunteering activities still helped those in need and answered religious prescriptions. The integration of market prescriptions with religious ones is perhaps well-exemplified in the volunteering activity of student mentorship, where high-performing students help others in need at disfranchised schools. A yearly internal review describes the activity as:

> encouraging the participation of those who stand out academically, building and expanding their role as citizens … By engaging as tutors, students use their expertise to serve others … By the end of the year, the tutor … receives a certificate from *Christ School*, formalizing their voluntary work—something increasingly important, especially in the context of those who intend to apply to foreign universities ... In addition, the student expands their socioemotional skills (Mentorship Project Report, *Christ School*)

The reconfiguration of resources associated with extracurriculars to integrate market prescriptions of developing soft skills and improving students' future employability and competitiveness for university admission ensures that the schools conform to market prescriptions. By integrating market prescriptions with religious ones, the schools also differentiate themselves from market-oriented competitors. Parents and students both recognize the unique added value of these volunteering extracurriculars. For example, parents value the simultaneous development of values and soft skills, which "teach the student to deal with real-life problems … [and to care] about relations between people, not just the content" (Joyce, parent, *Apostle School*). Students also acknowledge their development. For example, a 'student mentorship' participant testimonial (*Christ School*) mentioned how the student both realized his privilege and developed socioemotional skills. This student mentions how he "grew up a lot during my participation in volunteering activities because we had a great reality shock and we could see another side of our country's education ... and I understood that teaching is a great opportunity to learn."

Additional References

Moore, Phoebe, and Andrew Robinson (2016), "The Quantified Self: What Counts in the Neoliberal Workplace," *New Media & Society*, 18 (11), 2774–2792.

Web Appendix C: Strategies to Uniformize Responses to Marketization Across Organizations

Previous work on hybridization shows uneven hybridization patterns across different organizations (e.g., Pache and Santos 2013). Contrary to existing work, the five schools we analyzed present a strikingly similar trajectory. We emphasize five strategies that the BJEN and the schools used to facilitate a uniform adaptation to marketization across schools. We organize these strategies hierarchically, starting from top-management initiatives to school-level ones.

First, the organizations participated in collaborative planning. The BJEN, which includes members from each school, defined the objectives and priorities to adapt to marketization. It also created resources such as planning and implementation tools to achieve the goals that the schools established jointly. Collaborative planning ensured the buy-in from each school, as well as strategies that would fit their different realities.

The BJEN created multiple spaces for collaboration to facilitate joint planning across schools. These included the Council of Principals, which brings together the Principals from all schools of the network in periodic meetings to discuss objectives and priorities through a consensus-based decision-making process; Function-based committees, such as 'Curricula,' 'Continuous Teacher Education,' and 'Communications' committees, which bring together middle-level managers (e.g., teaching coordinators) from all schools to develop and find avenues to diffuse guidelines and implementation plans for function-related transformations; Task forces, or project-based committees, which also bring together middle-level managers from all schools to develop and find ways to diffuse guidelines and implementation plans for specific projects (e.g., developing the ENEM extracurricular); and an internal, online-mediated platform to collect opinions and ideas from employees of all levels and all schools. Collaborative planning fosters a sense of ownership regarding the changes to be implemented and strengthens managers' identification with changes to come—"a feeling of belonging to a network that is big, that is strong, that has a tradition" (Learning Coordinator, *Mary School*).

Take, for example, how the Educational Common Project (ECP), the main strategic document that the schools have been using to inform their responses to market prescriptions, was created. Its creation is explained in the document as follow (Introduction, arts. 16-18):

> During 2015, two task forces (TF) led the process of preparing the document. In the first semester, they held two seminars with the participation of more than 200 educators from all schools … Next, there were efforts to diffuse the findings devised during the workshops in each school. Those who participated in the seminars organized, along with upper management, spaces for socialization and conversation around the content, concerns, and propositions [of the ECP] … We broadened the invitation to participate to all employees (teachers and non-teaching staff) of the network. More than 2,000 professionals responded

> and participated in this exercise, which concluded the consultation and feedback stage [of the development of the ECP]. We then started a second task force to write the document.

Second, these collaborative planning activities led to the creation of documents and activities to generate information to support changes, facilitate the diffusion of information associated with responding to marketization, and explain how to implement transformations in practice. Examples of such documents include roadmaps and pamphlets that codify expectations, provide a course of action, and create evaluation metrics. These served to both manage and monitor efforts to transform the schools. These were widely diffused throughout the schools.

The network and the schools heavily relied on three instruments to support their transformation. The first is the Educational Common Project (ECP), a document composed of 117 articles that clarify the assumptions under which transformations are undertaken, codify the tri-partite educational philosophy, and propose routes for adaption as well as an implementation timeline. The "main objective [of the ECP] is to review, reposition and revitalize the apostolic work of the Society of Jesus in Education in Brazil and, at the same time, to inspire, guide, and direct the necessary adjustments" (Introduction).

The second is the School Quality Management System (SQSM). This document is grounded in "the principles of Jesuit pedagogy [and] studies on school efficiency" (SQMS, Introduction). It identifies four dimensions of Jesuit educational organizations central to providing an education of quality: curriculum; organization, structure, and resources; institutional climate; and families and the local community. It translates the Jesuit educational mission of providing holistic education into measurable variables. For each dimension, the System created guidelines for evaluation and expected goals, as well as stipulated how to implement evaluations to improve each dimension within the schools continuously.

The third is the National Market Research Project (NMRP), a comprehensive set of surveys conducted among parents, students, managers, and alumni of all schools of the BJEN to identify main value drivers and to evaluate the schools on specific dimensions related to schools' competitivity. The results of the NMRP provided information to assess and compare the strengths and weaknesses of the schools, which helped set up national and local priorities.

Third, to ensure the use of these documents and the adoption of guidelines at each school, the organizations performed 'tiered adoption.' By this, we mean that the schools worked at different organizational levels to facilitate transformations.

First, the schools created study groups and training programs to diffuse the ECP and other documents, which fostered their comprehension and adoption by managers and teachers. For example, at *Mary School*, "when the ECP arrived, we studied it in detail … we had meetings with the entire administrative area and meetings of smaller groups to study the route for implementation" (Giovanni, General Manager). These study groups helped managers understand

"what we teach, how we teach, and what we teach for" vis-à-vis "what we really want as a school [for example] in terms of holistic education" (Danton, Academic Director, *Apostle School*). The schools "encouraged teachers to study this document [the ECP] a lot. In all our memos, we have a citation from the ECP … each new teacher who comes in must study this document ... and we included it in our weekly [pedagogic] meetings." (Anelise, Academic director, *Priest School*). This equipped managers with the knowledge of the documents discussed in the previous strategy to implement changes within their school.

Each school also developed school-level roadmaps, such as learning maps, and workshop and courses to equip teachers with the expertise required to implement changes in day-to-day activities. Anelise explains:

> When faced with novelty and the unknown, we adapt with the support of our religious tradition … If we think about what schools, in general, are doing, something that is very interesting is the training of educators …. Everyone [at our schools] invests a lot in the training of educators. They meet weekly, for two or three hours, and in this training the question of innovation is always present [but we also do] an immersion in what is dear to us, which is our identity, to understand and discuss what is specific to education at a school of the Society of Jesus … Also, twice every semester … everyone gets the documents from the BJEN, which we read and then exchange around. There has never been, in these thirty years that I worked for the Society of Jesus schools, such a strong movement that I call dialectic [between] innovation, new methodologies, disruptive teaching [and the] Ignatian pedagogy, about our origin, our traditions.

Together, the centralized creation of guidelines through collaborative planning combined with tiered qualification created a chain of translation, from abstract goals to adapt to marketization at the strategic level (i.e., from the BJEN) to concrete, implementable, and measurable elements for each school, to day-to-day transformations by teachers. This not only provided clear guidelines for each organizational level, but it also equipped managers and employees to carry out transformations.

Fourth, the schools practiced resource reallocation. The BJEN used the SQMS and associated roadmaps, measurements, and feedback mechanisms and monitored the progress of the schools periodically to ensure a uniform progression. The BJEN developed several mechanisms to reallocate resources throughout schools to redress uneven developments. For example, investment priorities for each school were decided based on the results of the NMRP and SQMS. To ensure that each school had the financial resources necessary to implement the transformations, BJEN "created a fund to provide [financial] security to the schools [lacking financial resources]" (Father Santos, Top Manager, BJEN). Also, BJEN rotated high-level managers among schools to ensure the circulation of expertise. This was often done taking into consideration the success of each school in implementing transformations, i.e., where a member

of the upper management from a highly successful school would be rotated to a less successful one.

Fifth, in addition to these mechanisms that supported the diffusion and implementation of the guidelines created collaboratively by all members of the network, the BJEN also took responsibility for the implementation of some specific projects across the schools. Examples of such projects include a bilingual program, an enterprise resource planning (ERP) software, and a continuous education program for teachers and managers. This allows the network to benefit from economies of scale with third parties, as well as a uniform implementation across schools. Father Santos explains:

> The example of the bilingual program is good because the same process also occurred in other projects. So, the network assumed the responsibility of analyzing all the [potential service] providers to free [each] school from doing this negotiation and to ensure the same quality [from service providers for all schools]. So, we guarantee the same offer and the same opportunities. For this to happen, the network also takes care of negotiating the budget for the implementation of these projects to ensure a fair price for all schools.

In sum, in contrast to existing work that shows differences in hybridization patterns for the same hybridization strategy (e.g., Pache and Santos 2013), the schools transformed rather uniformly. We propose that uniformity resulted from two reasons. First, the schools we examined were all part of the same network, which contrasts with existing work that examined different organizations (e.g., Pache and Santos 2013). Second, the creation of the BJEN in 2014 was undertaken explicitly to address emerging challenges in the field of education in Brazil. As we showed, the network played an important role in ensuring a uniform transformation across schools.

Additional References

Pache, Anne-Claire, and Filipe Santos (2013), “Inside the Hybrid Organization: Selective Coupling as a Response to Competing Institutional Logics,” *Academy of Management Journal*, 56 (4), 972–1001.

Web Appendix D: Examples from Other Fields

Table 1. The Marketization of Museums (Nested market orientation)

Mechanism	Prescription	Outcome
Core Subsumption (Inverted towards the market)	Staging aesthetic exhibitions (Art); Staging profitable exhibitions (MKT)	High-profile market-oriented art-ified exhibitions, e.g., ‘The Art of the Motorcycle’ (BMW at the Guggenheim, 1998), ‘PUNK: Chaos to Couture’ (MET, 2013)
Core Integration	Educating the public (Art); Creating a customer experience (MKT)	Creation of a customer-oriented museum ‘experience’ (e.g., digitalization, immersive spaces, interactive spaces, Disneyfication, emphasizing the spectacular) (see Schubert 2016)
Peripheral Integration	Facilitating aesthetic experiences (Art); Attracting visitors (MKT); Contributing to a city’s international exposure (MKT)	Superstar architect creates museum expansion (e.g., Royal Ontario Museum in Toronto [Daniel Libeskind]) or museum building (e.g., Guggenheim Bilbao [Frank Gehry])
Peripheral Separation	Focus on profitability (MKT)	Hosting adult-only late-night events with alcohol and music (e.g., Royal Ontario Museum 'Friday Nights'; Art Institute of Chicago 'After Dark')
	Collecting art (Art)	Digitalizing a museum's core collection

Note: We concentrate on the logic of art and the market logic for this example.

Table 2. The Marketization of Hospitals (Nested traditional orientation)

Mechanism	Prescription	Outcome
Core Subsumption	Quality of care is determined by doctors (Care); Quality of care is determined by metrics (MKT)	Metrics (e.g., time to treatment; time to recovery) are used by doctors to improve quality of care (e.g., Foulkes 2011)
Core Integration	Care is governed by medical professions (Care); Care is governed by management (MKT)	Doctors assume hybrid roles (managerial and clinical) (Mendel and Scott 2010); Management effectiveness is based on clinical quality and performance, which recursively improves care (e.g., Tsai et al. 2015)
Peripheral Integration	Increased focused on customer experience (MKT)	Focus on overall 'patient experience,' strengthened through the optimization of service experiences, such as providing comfortable waiting areas, WiFi, clear parking signalization, and easy-to-use entrances (e.g., Berry, Carbone, and Haeckel 2002)
Peripheral Separation	Focus on profitability (MKT)	Creating a fine dining experience at hospital restaurants (Murphy 2017)
	Focus on care (Care)	Apps are used to maximize aftercare efficacy (e.g., Dash MD)

Note: We concentrate on the logic of care and the market logic for this example.

Additional References

These two examples were created by consulting the following work:

Agartan, Tuba I. (2012), "Marketization and Universalism: Crafting the Right Balance in the Turkish Healthcare System," *Current Sociology*, 60 (4), 456-471.

Berry, Leonard L., Lewis P. Carbone, and Stephan H. Haeckel (2002), "Managing the Total Customer Experience," *MIT Sloan Review,* April 15th, available at: https://sloanreview.mit.edu/article/managing-the-total-customer-experience/.

Boorsma, Peter, Annemoon Van Hemel, and Niki Van Der Wielen (eds.) (1998). *Privatization and Culture: Experiences in the Arts, Heritage, and Culture Industries in Europe.* Dordrecht: Springer.

Bradford, Gray H. (1985), "An Introduction to the New Health Care for Profit," *in The New Health Care for Profit: Doctors and Hospitals in a Competitive Environment,* Institute of Medicine, National Academies Press.

Cribb, Alan (2008), "Organizational Reform and Health-care Goods: Concerns about the Marketization in the UK NHS," *Journal of Medicine and Philosophy,* 33, 221-240.

Dunn, Mary B. and Candace Jones (2010), "Institutional Logics and Institutional Pluralism: The Contestation of Care and Science Logics in Medical Education, 1967-2005," *Administrative Science Quarterly,* 55, 114-149.

Ekström, Karin (ed.) (2020). *Museum Marketization: Cultural Institutions in the Neoliberal Era.* London: Routledge.

Foulkes, Mark (2011), "Nursing Metrics: Measuring Quality in Patient Care," *Nursing Standard,* 25 (42), 40-45.

Frey, Bruno (S.) (1998), "Superstar Museums: An Economic Analysis," *Journal of Cultural Economics,* 22 (3/4), 113-125.

Mol, Annemarie (2008). *The Logic of Care: Health and the Problem of Patient Choice.* London: Routledge.

Murphy, Brooke (2017), "Some Hospitals are Getting Into Fining Dining—Here's Why," *Becker's Hospital Review,* April 19th, available at: https://www.beckershospitalreview.com/capital/some-hospitals-are-getting-into-fine-dining-here-s-why.html.

Porter, Michel E. and Thomas H. Lee (2013), "The Strategy that Will Fix Health Care," *Harvard Business Review,* October, available at: https://hbr.org/2013/10/the-strategy-that-will-fix-health-care.

Schubert, Karsten (2009). *The Curator's Egg: The Evolution of the Museum Concept from the French Revolution to the Present Day.* London: Ridinghouse.

Scott, W. Richard, Marin Ruef, Peter J. Mendel, and Carol A. Caronna (2000). *Institutional Change and Healthcare Organizations: From Professional Dominance to Managed Care.* Chicago: University of Chicago Press

Tsai, Thomas C., Ashish K. Jha, Atul A. Gawande, Robert S. Huckman, Nicholas Bloom, and Raffaella Sadun (2015), "Hospital Board And Management Practices Are Strongly Related To Hospital Performance On Clinical Quality Metrics," *Health Affairs,* 34 (8), 1304-1311.
